# Snakebite in the Hills and Mountains of Nepal

**DOI:** 10.31729/jnma.7562

**Published:** 2022-12-31

**Authors:** Sunil Bogati, Navin Bhatt, Shristi Nepal, Prakash Nepali, Sanjib Kumar Sharma

**Affiliations:** 1 Kolhabi Primary Health Care Center, Kalaiya, Bara, Nepal; 2 Tribhuvan University Teaching Hospital, Institute of Medicine, Maharajgunj, Kathmandu, Nepal; 3 Bhimad Primary Health Care Center, Tanahun, Nepal; 4 Department of Internal Medicine, B.P. Koirala Institute of Health Sciences, Dharan, Sunsari, Nepal

**Keywords:** *antivenom*, *Nepal*, *snakebite*

## Abstract

Snakebite is an important public health issue around the world. In Nepal, it affects a huge number of people mostly belonging to low-income households who are involved in agriculture. Although snakebite has a serious impact on the Terai population, a few studies suggest that snakebite also occurs frequently in hills and mountains. In the absence of sufficient studies related to snakebites in these geographical regions, it is tough to sketch a true picture and estimate the magnitude of snakebites in those areas. Because of this, the healthcare system is probably not prepared enough to handle the victims of snakebites. This demands a proper study of the burden of the issue in these regions and appropriate initiatives for addressing it.

## INTRODUCTION

Hot and humid days come with the looming danger of snakebites around the world. Snakebites are one of the most important tropical public health problems.^1^ Every year, around 5.5 million snakebites occur worldwide, resulting in 421,000 envenomation and 20,000 deaths.^2^ Despite the fact that 6.85 billion people live in snake-infested places, roughly 2.14 per cent live in remote areas without access to appropriate health care.^2^ In Nepal around 40,000 individuals are reported to be bitten by snakes each year, with nearly 3000 people dying as a result.^3^ The problem is most common among people from low-income households who are engaged in agriculture in rural areas of the Terai. Around 17 snake species have been found to be venomous among Nepal's 89 snake species, mostly belonging to the Elapidae and Viperidae families.^4^ Nearly 10 of the venomous snake species can be found in the hilly and mountainous regions.^4^ The majority of snakebite deaths go unreported, and there is no precise data on the morbidity and mortality of snakebite patients in Nepal's hills and highlands; much has to be explored.^5,6^

## TOPOGRAPHIC VARIATIONS OF SNAKEBITE

Nepal is a landlocked country with three ecological divisions: the Terai, the Hills, and the Mountains.^7,8^ Before the recent nationwide survey in the Terai region of Nepal,^3^ there was no nationally representative data on snakebites in Nepal. Unfortunately, the recent epidemiological study did not include the population from the hills and mountains.^1^ Terai is the southernmost part of the country at an altitude of 60 to 305 meters.^7^ With more than half of the population living in Terai and people mostly engaged in agricultural activities (65%), this region has the greatest human and animal snakebite incidence, with an estimated 261 incidences per 100,000 people each year.^3^

The hilly region is the part at an altitude above 610 meters that comprises the largest land area of the country, and it has 43.1 per cent of the total population.^7^ The mountainous region is the northernmost part, with an altitude above 4877 meters, and is the least populated ecological division.^9^ Studies pertaining to snakebites in hills and mountains are scarce. Some of the highly venomous snake species like Monocellate cobra *(Naja kaouthia),* Himalayan krait *(Bungarus bungaroides),* Greater black krait *(Bungarus niger),* King cobra *(Ophiophagus hannah*), Himalayan pit viper *(Gloydius himalayanus),* Tibetan pit viper *(Himalayophis tibetanus),* Mountain pit viper *(Ovophis monticola),* Himalayan Habu pit viper *(Protobothrops* sp.), White lipped pit viper *(Trimeresurus albolabris)* and Kramer's pit viper *(Trimeresurus septentrionalis)* are found in the hilly and lower mountain regions.^4^

## CLINICAL OUTCOMES OF SNAKEBITES IN HILLS AND MOUNTAINS OF NEPAL

The green pit vipers and mountain pit vipers are widely distributed and frequently encountered venomous snake species in the hills and mountains of Nepal.^4,10^ The common symptoms reported due to snakebite in these regions include local site pain, swelling with local tissue damage, and haematological features like coagulopathy, hematuria and thrombocytopenia.^11-13^ Attempts to manage these symptoms have been done with Government supplied polyvalent antisnake venom for venom neutralization, vitamin K and Fresh Frozen Plasma (FFP) injection for coagulopathy management, MgSO_4_ dressing and antibiotics for local manifestations of the snakebite and solely conservative management has been done at the centres with no availability of anti-snake venom.^11-13^ Complications like severe coagulopathy, intrauterine foetal demise, and renal failure have been reported in studies from other countries.^14,15^ Although rare, symptoms like ptosis, respiratory distress, drooling, and coma suggestive of neurotoxic envenomation were reported in a patient in the Hilly region.^12^ Because of the limited availability of good published studies, it is hard to comment on the complications and mortality of snakebites in the hills and mountains of Nepal.

## CHALLENGES IN THE MANAGEMENT AND ACCESS TO CARE

The challenge in the management of snakebites is highly influenced by the perception of people living there. People's perceptions influence the state or time in which a patient reaches the hospital for treatment. People's awareness of snakebites determines what measures they resort to as first aid. Most people feel the need for a tourniquet, soap water, mud application at the bitten sites and incision/drainage as the first aid to any snakebite case.^16^ In remote villages of Nepal conventional methods of treatment like reading religious chants, attempting to suck venom using the anal sphincter of a chicken, lexins, and latex from snake guard plants are famous.^17^ Using such methods mostly causes deterioration of the symptoms of patients. Some patients who worsen during the course of treatment under the faith healers, get referred to hospitals when it is quite late, resulting in the death of patients during transit.^5,6^

Most snakebite deaths go under-reported owing to the low number of patients reaching the health facilities.^5,6^ Even when patients reach the hospital, there is a barrier of less doctor-patient ratio,^18^ and inadequate knowledge and skills needed for approaching a snakebite patient among the health care providers.^17^ Most physicians in South Asia have to rely on the circumstances of the bite and clinical features of envenomation to find out the envenoming species of snake.^19^ There is a lack of appropriate anti-snake venom, accessory medications, and blood products, along with a shortage of critical care equipment and infrastructure at the health centres in the Hills which makes referrals to a higher centre a necessity.^20^ No antivenom is available to treat pit viper envenoming as there is no evidence that the polyvalent snake antivenoms imported from India neutralize the venoms of other species of snakes in Nepal.^4,21^ Often, the referral centres are far away, with tedious transportation routes,^22^ making it even more troublesome to reach a good facility. Even if some patients reach referral centres, the long transit time raises the probability of local and systemic complications, sometimes resulting in death on the way to the hospital. The logistic challenges that follow the treatment at referral centres deter many victim families from even considering this option, as the majority of snakebite patients belong to low-income families.

## PREVENTION AND MANAGEMENT

The snakebite envenoming working group of WHO has developed a strategic road map on snakebites which aims to reduce mortality and disability due to snakebite envenoming by half by 2030.^1^ Empowering and engaging communities is the first task to be done. People should be educated about the local species of snakes, their habitats, their interaction with humans and ways to prevent getting bitten. Timely access to the proper medical care needs to be emphasized. The rampant use of conventional first aid methods in villages can be reformed with education regarding the correct first aid measures via different public communication platforms relevant to rural settlements. As faith healers have a revered status among the rural localities, they can be approached with awareness programs regarding the reality of snakebites and their role in encouraging people to go to health centres promptly. Ensuring safe and effective treatment needs to be done. This is possible when the health care staff in the hills and mountains are properly trained regarding the approach to a snakebite patient. Also, the provision of required antivenom, resuscitation equipment, and other accessories needs to be done at the health centres. As we are heavily reliant on polyvalent anti-snake venom prepared against the great four snakes, endeavours should be made to study the possible role of targeted anti-snake venom for the common snakes in the Hills like the Green pit viper and Mountain pit viper. When required, patients need to be referred to the nearest tertiary care centre with appropriate first-hand management of their case. The third thing is the strengthening of health systems. As the patient population is made aware to seek health care at health centres, it is imperative to prepare all sorts of human and physical resources at the centres so that the concerns of patients can be addressed well, and the best possible treatment can be provided to the patients. Telemedicine services can be utilized by the circulation of images of envenoming species of snakes via the media like WhatsApp, Viber or Messenger from the healthcare personnel to the experts, which can ease the delivery of focused care to the victim in rural health centres. Awareness must be spread among the residents of Hills regarding the need for notification to the health authorities in case of any snakebite. In addition, the staff at health centres must be trained in the Central Data Management System, which enables the accumulation of unified data on snakebite, which helps to devise targeted plans at the government level in succession. Lastly, it is a must to increase partnerships, coordination, and resources. Snakebite in hills and mountains is a problem that can be resolved with the combined efforts of the government and nongovernmental organizations from both the national and international community, along with local people and other stakeholders. The concerned authorities should be encouraged to take the lead on the task of improving transport facilities so that patients referred to the higher centres can reach their destination on time. Efforts should be made to address the issue at the policy level too, by allocating budgetary resources targeted towards snakebite management at health centers.

## WAY FORWARD

A study regarding the actual burden of snakebites in hills and mountains is a necessity. Formulation of protocols for the treatment of the patients keeping in mind the differences in the types of snakes commonly encountered in these geographical areas compared to the plains of Nepal would be a better option. After a framework of the approach is made, it can be implemented with coordination among the people, government, and non-governmental organizations.
